# Brain Dynamics of Action Monitoring in Higher-Order Motor Control Disorders: The Case of Apraxia

**DOI:** 10.1523/ENEURO.0334-20.2021

**Published:** 2022-03-02

**Authors:** Giuseppe Spinelli, Rachele Pezzetta, Loredana Canzano, Emmanuele Tidoni, Salvatore Maria Aglioti

**Affiliations:** 1 IRCCS Santa Lucia Foundation, Rome 00179, Italy; 2 myBrainTechnologies, Paris 75010, France; 3 IRCCS San Camillo Hospital, Venice 30126, Italy; 4 Human Technology Laboratory, University of Hull, Dep. of Psychology, Cottingham RD, Hull, HU6 7RX, United Kingdom; 5 Sapienza Università di Roma, 00185 and cn2ls@sapienza, Istituto Italiano di Tecnologia, 00197, Italy; 6 Department of Neurorehabilitation, Bambino Gesù Children’s Hospital, Rome, 00050, Italy

**Keywords:** apraxia, EEG, performance monitoring, theta oscillations, virtual reality

## Abstract

Limb apraxia (LA) refers to a high-order motor disorder characterized by the inability to reproduce transitive actions on commands or after observation. Studies demonstrate that action observation and action execution activate the same networks in the human brain, and provides an onlooker’s motor system with appropriate cognitive, motor and sensory-motor cues to flexibly implementing action-sequences and gestures. Tellingly, the temporal dynamics of action monitoring has never been explored in people suffering from LA. To fill this gap, we studied the electro-cortical signatures of error observation in human participants suffering from acquired left-brain lesions with (LA+) and without (LA–) LA, and in a group of healthy controls (H). EEG was acquired while participants observed from a first-person perspective (1PP) an avatar performing correct or incorrect reach-to-grasp a glass action in an immersive-virtual environment. Alterations of typical EEG signatures of error observation in time (early error positivity; Pe) and time-frequency domain (theta band-power) were found reduced in LA+ compared with H. Connectivity analyses showed that LA+ exhibited a decreased theta phase synchronization of both the frontoparietal and frontofrontal network, compared with H and LA–. Moreover, linear regression analysis revealed that the severity of LA [test of upper LA (TULIA) scores] was predicted by mid-frontal error-related theta activity, suggesting a link between error monitoring capacity and apraxic phenotypes. These results provide novel neurophysiological evidence of altered neurophysiological dynamics of action monitoring in individuals with LA and shed light on the performance monitoring changes occurring in this disorder.

## Significance Statement

Combining EEG and immersive virtual reality we provide novel neurophysiological evidence of altered performance monitoring in apraxic patients. We show that the observation of incorrect actions performed by an avatar seen from a first-person perspective (1PP) elicited reduced electrocortical markers of error detection in apraxic patients. Tellingly, apraxia severity predicted reduction of mid-frontal theta activity, regardless of brain lesion volume and patients’ cognitive capacity. The results shed new light on the possible neurophysiological signatures of the link between limb apraxia (LA) and performance monitoring. Moreover, our EEG-virtual reality paradigm may provide a new tool for investigating the brain dynamics of monitoring action errors also in brain damaged patients with motor limitations.

## Introduction

Limb apraxia (LA) is a disorder of higher order motor control mainly associated with damage of left frontoparietal brain networks (Buxbaum et al., 2014; Buxbaum and Randerath, 2018; Bizzozero et al., 2000). LA is characterized by a complex combination of perceptual (Halsband et al., 2001), motor (Candidi et al., 2017), and cognitive (Rothi et al., 1985) deficits whose interaction ultimately affects the implementation of transitive and intransitive movements on verbal command or imitation. According to the “affordance competition hypothesis” (Cisek, 2007), potential actions compete against each other, and information is collected to bias and solve this competition until a response is selected. Competition arises from mere sensory exposition to an object and its physical properties that automatically triggers conflicting action schema for “affording” the object itself (Cisek, 2007; Cisek and Kalaska, 2010), and may lead to performance errors if the conflict is not resolved (Cooper, 2007). Tellingly, apraxic patients not only display deficits in action execution but also in action understanding and simulation (Rothi et al., 1985; Cubelli et al., 2000), in mental action imagery task (Sirigu et al., 1999), in generating internal models for action execution (Buxbaum et al., 2005), and in the judgment of the correctness of seen or heard (Pazzaglia et al., 2008a, b; Aglioti and Pazzaglia, 2010, 2011; Canzano et al., 2014) actions. Moreover, deficits in action monitoring were positively correlated with difficulties in action execution (Pazzaglia et al., 2008a) thus, corroborating the hypothesis of a direct matching between action perception and execution. In line with the affordance competition hypothesis, studies suggest that errors in apraxia could be because of a deficient resolution of competition between action selection (Jax and Buxbaum, 2013; Buxbaum et al., 2014; Watson and Buxbaum, 2015) or to a failure to resolve affordance competition (Rounis and Humphreys, 2015). In keeping with Bekkering et al. (2000), when an action is observed, it is the action goal that is observed, and not just a movement. Action observation and execution are bidirectionally linked, so that motor skills may improve as an effect of merely seeing others moving (Ertelt et al., 2007; Cross et al., 2009; Ernst and Steinhauser, 2017). Moreover, performing specific actions improves the ability to perceive them (Casile and Giese, 2004; Lepage and Théoret, 2006). Monitoring actions through observation implies the evaluation of their correctness. EEG studies demonstrate that observation of errors in one’s own and others’ actions elicits specific markers over the mid-frontal cortex, namely, (1) the observer error-related negativity (oERN), the observer error positivity (oPe; van Schie et al., 2004; de Bruijn et al., 2007; Ridderinkhof et al., 2009), and (2) increased power in the theta band (4–8 Hz; Cavanagh et al., 2009, 2010, 2012). These patterns of electro-cortical brain activity are likely associated to conflict processing and resolution (Cavanagh and Frank, 2014). Conflict arises when a unique (correct) action should be selected among a set of competing (incorrect) actions and serves as an alarm signal conveyed from the mid-frontal to the lateral prefrontal and posterior brain areas to increase cognitive control over actions (Steinhauser and Yeung, 2010; Cohen and Cavanagh, 2011; van Driel et al., 2012; Yoshida et al., 2012; Boldt and Yeung, 2015).

The present study aims to investigate the temporal dynamics of action monitoring in patients suffering from LA by linking the “affordance competition theory” and the “conflict monitoring model.” Crucially, both theories consider conflict processing as a fundamental mechanism by which the performance monitoring system exerts motor and cognitive control over actions. In view of the affordance-competition hypothesis, we predict that patients with LA tend to experience high levels of conflict during goal-directed action monitoring, which arises from the competition between correct and incorrect action schemas. This may lead to an exaggerated burden of unresolved conflict that impairs the operation of the action monitoring system. Capitalizing on previous similar reports (Pavone et al., 2016; Pezzetta et al., 2018; Spinelli et al., 2018), we recorded EEG in left-brain damaged individuals with and without LA and in a control group who observed through immersive virtual-reality an avatar performing correct or incorrect actions. In line with previous studies on error monitoring, awareness, and gesture recognition in patients with apraxia (Canzano et al., 2014, 2016; Candidi et al., 2017; Scandola et al., 2021), we expected an impairment in patients with LA when the error monitoring system is called into play, that is when a mismatch between predicted and observed action goal occurs. Acquiring EEG signatures of performance monitoring during the observation of correct and incorrect actions provided novel information on the integrity of the error detection system in LA.

## Materials and Methods

### Participants

Twelve right-handed, left-brain damaged patients were recruited from the local Neuro-Rehabilitation Unit between March and August 2016. They had suffered from focal vascular lesions (e.g., patients with traumatic brain injuries were not included) between 292 and 1095 d (LA+: mean = 580.33; SD = 252.48; LA–: mean = 687.17, SD = 207.08); thus, they were tested during chronic phase (Karnath and Rennig, 2017). A primary inclusion criterion was the ability to perform the task (EEG-VR session), and to understand the task instructions. All the participants signed an informed consent for participation. Based on a neuropsychological assessment (Table 1) of their symptoms, participants were divided in two groups: (1) patients with (LA+; *n* = 6, 4 males and 2 females) and (2) without (LA–; *n* = 6, 3 males and 3 females) LA. The two groups were matched for age (mean age ± SD: LA+ = 63.1 ± 14.4 years, LA– = 58.5 ± 14.2 years) and education (LA–: 12 ± 2.0; LA+: 13.8 ± 3.4). An age-and-gender-matched (mean age ± SD: 62.4 ± 11.2, 6 males, 4 females) sample of 10 healthy participants (H) was also tested. An age-and-gender matched (mean age ± SD: 62.4 ± 11.2) sample of 10 healthy participants (H) was also tested. The study was conducted in accordance with the guidelines of Declaration of Helsinki and approved by the local Ethics Committee.

**Table 1:** Demographic and clinical data

Participant	Age (years)	Education (years)	Interval from lesion (days)	Raven (10 min)	TULIA	Apraxia of utilization	Word comprehension	Sentence comprehension	FAB tot 3–6 (mean)	Line bisection
LA-1	70	13	563	32.5	222	14	30	28	3	9
LA-2	41	18	531	30	228	14	28	30	2.7	9
LA-3	63	13	627	29.5	231	14	30	24	3	9
LA-4	39	13	619	27	228	14	29	26	3	8
LA-5	51	13	688	32	234	14	30	30	3	9
LA-6	80	13	1095	26	228	14	30	22	3	9
Mean (±SD)	57.33 (16.43)	13.83 (2.04)	687.17 (207.08)	28.75 (2.75)	228.5 (3.99)	14 (0)	29.5 (0.83)	26.67 (3.27)	3 (0)	8.83 (0.41)
LA + 1	70	8	473	16.5	127	12	20	25	2	9
LA + 2	80	13	532	16.5	137	14	28	19	1	7
LA + 3	68	17	498	31.5	180	14	27	13	2	8
LA + 4	33	13	648	31.5	165	14	26	26	3	9
LA + 5	78	8	292	24.5	162	14	23	17	2	8
LA + 6	68	13	1039	24.5	192	14	30	25	2	9
Mean (±SD)	66.17 (17.05)	12 (3.46)	580.33 (252.48)	24. 17 (6.69)	160.5 (24.78)	13.67 (0.82)	25.67 (3.61)	20.83 (5.31)	2 (0.63)	8.33 (0.82)
*Z*-score	−0.800	0.880	−2.081	1.601	2.882	0.48	2.161	1.841	2.321	1.04
*p*-value	0.423	0.378	0.037*	0.109	0.003*	0.630	0.03*	0.06	0.02*	0.297

All patients are in their chronic stage according to Karnath and Rennig (2017). Asterisks indicate significance between groups (Mann–Whitney *U* test).

In order to inform on the patients’ cognitive profile, standard tests and batteries for general neuropsychological assessment were administered (for details, see Table 1), including general cognitive abilities (Raven et al., 1988), executive functions (nonverbal subtests of the frontal assessment battery; Appollonio et al., 2005), and spatial attention (line bisection; Wilson et al., 1987). Verbal comprehension and denomination subtests of the Aachener aphasia test (Luzzatti et al., 1996) were used to assess language comprehension deficits. Given that the experimental task implied the mere observation of correct versus erroneous upper limb actions, the assessment of apraxia focused on tests where actions implied the use of upper limbs, namely, the ideomotor [test of upper LA (TULIA); Vanbellingen et al., 2010], and the ideational apraxia tests (De Renzi and Lucchelli, 1988). The two groups did not differ in ideational apraxia (see Table 1), suggesting that semantic knowledge concerning actions was preserved. While LA+ presented difficulty in understanding words with respect to LA–, no such effect was found for sentence comprehension. This result, together with the nature of the task, suggests that comprehension did not play a major role in the experimental effects.

Analysis of brain lesions was conducted for LA– and LA+ by means of the MRIcron software (https://www.nitrc.org/projects/mricron; Rorden and Brett, 2000). The MRI/CT scans available for all the patients were mapped by drawing on the standard T1-weighted MRI template (ICBM152) of the Montreal Neurologic Institute (MNI) coordinate system, approximately oriented to match the Talairach space (Talairach, 1988). The standard template (size: 181× 217 × 181 mm, voxel resolution: 1 mm^2^) was rotated on the three planes to match each patient’s MRI/CT scan orientation as closely as possible. Then, two experienced clinicians (who were blind as to which patients the scan belonged to) traced any lesion manually on the axial slices of the rotated template, while another one checked all the drawings in a double-blind procedure (de Haan and Karnath, 2018). For each patient the outcome was a map of the damaged areas with each voxel labeled as 0 (intact) or 1 (lesioned). All the lesion maps were rotated back to the canonical orientation to align them to the standard stereotaxic MNI space (in 2 × 2 × 2 mm voxel). After that, maps were filtered with a custom mask based on the ICBM152 template to exclude the voxels of lesions outside the white and gray matter brain tissues. Each patient’s lesion was superimposed onto T1 templates to calculate the number of lesioned voxels in various cerebral areas, and the center of the mass of each damaged area. This was then overlapped onto the automatic anatomical labeling (AAL) template (Tzourio-Mazoyer et al., 2002) to provide information on the gray matter, and onto the JHU white-matter atlas (Susumu Mori, Laboratory of Brain Anatomical MRI, Johns Hopkins University) for the white matter. LA+ and LA– lesion overlap and lesion subtraction were performed to highlight patients’ lesional patterns. For each region, the MNI coordinates of the center of mass along with the number (*n*) and percentage (%) of clustering voxels are provided for LA+, LA–, and subtraction lesion map (Tables 4, 5). Analysis of tract disconnection probability were also conducted, by mapping the lesion from each patient onto tractography reconstructions of white matter pathways obtained from a group of healthy controls (Rojkova et al., 2016). We quantified the severity of the disconnection by measuring the probability of specific tracts (Thiebaut de Schotten et al., 2014) using Tractotron software as part of the BCBtoolkit (Foulon et al., 2018; http://www.toolkit.bcblab.com; Table 6). We computed *t* test comparison with false discovery rate correction to verify significant differences between groups.

### Apparatus and virtual environment

Participants were seated in a four screens (3 × 3 × 2.5 m) cave automatic virtual environment (CAVE) system (Cruz-Neira et al., 1993; Fig. 2*A*). 3D images were alternatively eye-by-eye displayed at a refresh rate of 60 Hz by Nvidia Stereo Glasses, which were in turn interfaced with an Intersense 900 ultrasonic system (Thales Visionix; 6 degrees of freedom). The virtual scenario included a virtual room (3 × 3 × 2 m) with a virtual table, and an avatar with both its right (R) and left (L) upper limb on the table (Fig. 2*B*). Atop the table was a yellow support with the virtual glass placed on it. The virtual scenario and the avatar were drawn on a 1:1 scale by Maya 2011 and 3ds Max 2011 (Autodesk, Inc) respectively, and rendered by XVR 2.1 (Tecchia et al., 2010). The avatar’s kinematics were implemented using Halca libraries (Gillies and Spanlang, 2010). Marker events were sent to the EEG by means of a custom-made circuit governed by a digital input/output device (PoKeys 55; PoLabs; https://www.poscope.com).

### Experimental design

**Figure 1.**
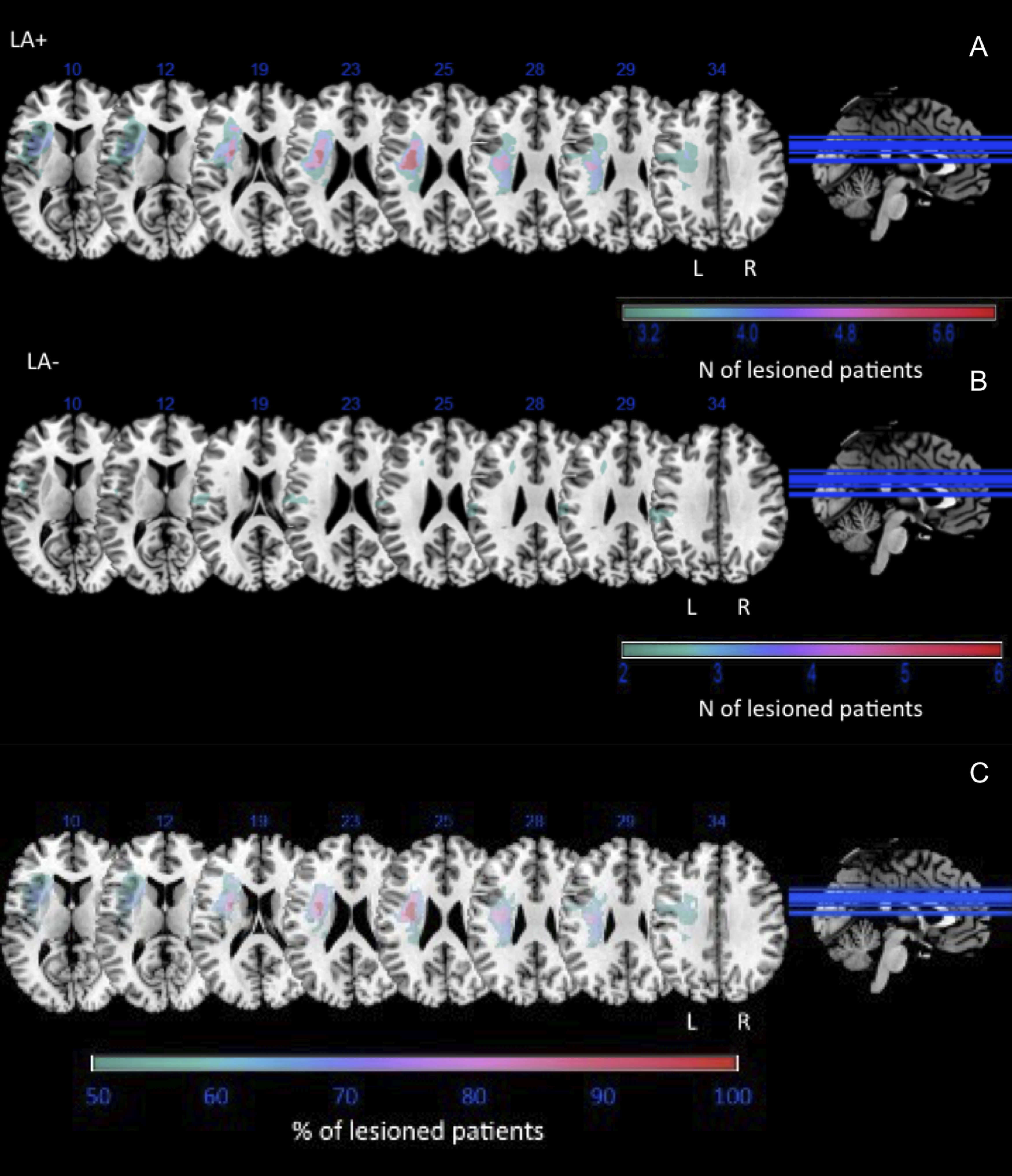
Brain lesion analysis. Color rendering of the lesion patterns in patients with limb apraxia (LA+; panel ***A***), patients without limb apraxia (LA–; panel ***B***) and LA+ minus LA– subtraction (panel ***C***). The figure shows the typical pattern of frontoparietal damage typical of apraxia.

Expanding on previous reports (Pavone et al., 2016; Pezzetta et al., 2018; Spinelli et al., 2018), the main task used in this study implied that participants observed correct or incorrect reach-to-grasp a glass actions performed by an avatar seen from a first-person perspective (1PP). Participants were immersed in the virtual scenario and their physical body was aligned with the virtual body to maximize embodiment. The participants’ real body was occluded by a black cloth. Each trial started with an intertrial interval (ITI) lasting 1250 ms (±250 ms) in which both avatar’s upper limbs rested on the table. After a synthesized voice instructed the avatar to grasp the glass (2000 ms), participants observed one of the two avatar’s limbs (R or L, depending on the experimental block) reaching and grasping the virtual glass (Fig. 1*B*). Each reach-to-grasp action lasted 1000 ms, such that the first 700 ms were identical for all actions, and the last 300 ms defined a correct (C) or incorrect (I) outcome. While correct actions resulted in a successful grasping of the virtual glass, incorrect actions depicted a virtual limb directed five-virtual-centimeter right-ward the virtual glass (or five virtual-cm left-ward in the case of left arm movements). A total of 2000 ms elapsed after the completion of each action, before the virtual limb returned to its starting position. The whole experiment counted 120 trials, split in two blocks of 60 trials, each containing R or L avatar’s actions exclusively. The order of blocks (R and L) was counter-balanced within participants for each group (LA+, LA–, and H). Correct (*n* = 36) and incorrect (*n* = 24) actions were randomly presented across the trial-list of each block.

**Figure 2.**
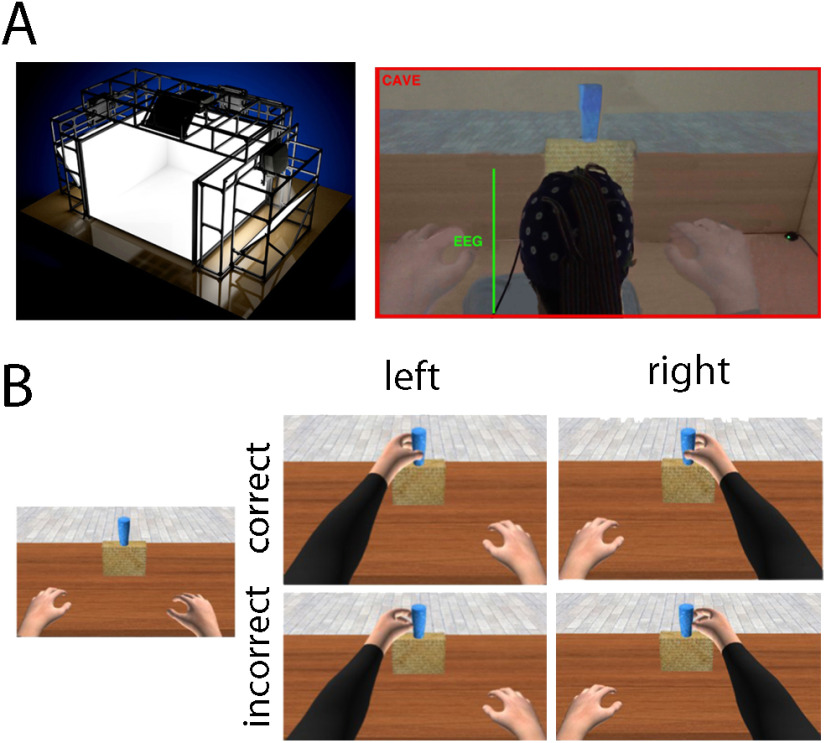
Apparatus and experimental task. ***A***, Four-screen CAVE system (left) and a snapshot of an actual experimental trial (right) depicting a participant seeing a virtual limb from 1PP during the EEG recording. ***B***, Rendering of the virtual scenario as seen from the 1PP. The avatar has its own upper limbs placed on the table at ∼50 cm from the virtual glass (left). On the right side, combinations of avatar’s action outcomes that participants observed in the four experimental conditions [ACCURACY (correct, incorrect) × LIMB (right, left)].

Subjective ratings of virtual embodiment (i.e., sense of ownership and vicarious agency) were collected in the 25% of trials (i.e., 30 trials). Participants were asked to separately rate on two visual-analog scales (VASs): (1) how strongly the virtual arm was felt as part of their body (feeling of ownership; Ow), and (2) how much they felt in control of the virtual arm (feeling of Vicarious Agency; Ag). Ratings were acquired at the end of avatar’s actions, by asking participants to quantify the strength of their feelings by positioning a virtual stick on the VAS ranging from 0 to 100, where 0 indicated “no feeling” and 100 “highest feeling.” The different VASs were sequentially displayed on a black box appearing ahead the virtual glass and disappearing immediately after an answer was provided. Each participant provided a total of 15 self-reports of Ow and Ag in each block, nine for C and six for I. The order of Ow and Ag self-reports was counter-balanced across trials.

### EEG recording and analysis

EEG data were acquired by means of tin electrodes embedded in a fabric cup (Electro-Cap International), according to the 10–10 system, from 60 scalp sites (Spinelli et al., 2018). The electrode on the right earlobe served as online reference, while the ground electrode was placed on AFz. A bipolar electro-oculogram was recorded from two electrodes placed on the lateral end of the bicanthal plane. The signal was recorded by a Neuroscan SynAmpsRT (Compumedics, Ltd) at 1000 Hz, and filtered with a hardware bandpass of 0.05–200 Hz. All impedances were kept below 5 KΩ. EEG traces were processed using the FieldTrip toolbox (Oostenveld et al., 2011; release: 20170607) in MATLAB R2016a (The MathWorks).

For each subject, continuous EEG signals were filtered offline with a 0.5-Hz high-pass FIR filter (onepass, zero-phase) and locked to the onset of the avatar’s arm-path deviation (i.e., 300 ms before action-offset). This time point corresponded to the latest timeframe in which observed grasping trajectories were still identical between correct and incorrect actions (Spinelli et al., 2018). Epochs of 6 s (±3 s around this trigger) were extracted and sorted according to the ACCURACY of the observed avatar’s action (two levels: C and I), and to the avatar’s LIMB that was observed (two levels: R and L). Blinks and oculomotor artifacts were removed by the independent components analysis (ICA). On average, 3.6 components (range: 1–7) referring to blink/oculomotor artifacts were discarded. Trials exhibiting residual artifacts were discarded by means of (1) a summary plot of three metrics (variance, *z* score, kurtosis) of all channels, as implemented in FieldTrip, and (2) a further visual inspection of all segments and all channels. Details of remaining trials are shown in Table 2. The obtained artifact-free time series were then re-referenced to the common-average reference and baseline corrected with respect to a time window of 200 ms before the trigger (i.e., the onset of avatar’s arm-path deviation). Time- (Event Related Potentials, ERPs), time-frequency (TF) domain and phase connectivity analyses were conducted.

**Table 2:** Trials count after artifact-rejection

	Right		Left	
	Correct (out of 36)	Incorrect (out of 24)	Correct (out of 36)	Incorrect (out of 24)
LA+ (mean; %; range)	33.0 (92%); 32–34	23.0 (96%); 22–24	34.3 (95%); 33–36	23.3 (97%); 20–26
LA– (mean; %; range)	35.0 (97%); 33–36	23.6 (97%); 22–24	34.9 (97%); 33–36	23.5 (97%); 22–24
H (mean; %; range)	34.0 (94%); 28–38	22.8 (95%); 18–27	34.0 (94%); 28–38	22.7 (95%); 18–27

Results are shown for each group (patients with limb apraxia, LA+; patients without limb apraxia, LA– and healthy controls, H) and condition (right/left × correct/incorrect).

For ERPs analysis, the across-trials average for each condition [LIMB (R, L) × ACCURACY (C, I)] was obtained in the time range of −200–800 ms. This time window was considered for statistical analyses. TF analysis was conducted by means of the wavelets method. Width (or cycles) of each wavelet was 4 (i.e., 4/2πf). Frequency resolution was 1 Hz (range: 4–30 Hz). Length of the time window for computation was 2.6 s (±1.3 s around the trigger). Time-resolution was 50 ms. TF spectra were corrected to the relative signal change (% change) of the event period (from 0 to 1000 ms) with respect to the baseline (from −200 to 0). The average across trails for each condition was calculated in the time window from −200 to 1000. This time window was used for statistical analyses. Functional connectivity analysis was conducted by computing the trial-by-trial phase locking value (PLV; Lachaux et al., 1999) for across channels combinations. Imaginary coherence was considered to compensate for volume conduction issues (Vinck et al., 2011). Oscillatory phase synchronization between channels is considered a connectivity measure that reflects the exchange of information between neuronal populations (Sauseng and Klimesch, 2008).

### Statistical analysis

In order to statistically estimate time- and time-frequency differences between groups (LA+ vs LA– vs H) and within conditions (LIMB and ACCURACY) at each electrode, a nonparametric Monte Carlo permutation was conducted (1000 repetitions). As first, a permutation distribution of the significance probabilities for dependent-samples *t* tests between R versus L was calculated separately for each group. Since no significant results were obtained (all *p* > 0.05), voltage/power values of both conditions (R and L) were averaged. On these obtained time-series, dependent-samples *t* tests were conducted to estimate the differences between C versus I separately for each group using nonparametric cluster-based permutation analysis as implemented in Fieldtrip (cluster-α = 0.05). Contrasts between groups were computed by means of three independent-samples *t* tests (H vs LA+, H vs LA–, LA– vs LA+) using voltage/power values difference between incorrect and correct conditions (I minus C). To correct for multiple comparisons, a cluster-based correction was applied to all tests as implemented in FieldTrip (cluster-α = 0.05; Maris and Oostenveld, 2007).

Like for ERPs and TF analyses, PLV values of the condition LIMB (L and R) were averaged as no difference was found (*p* > 0.05). Transient theta phase activity from mid-frontal to lateral prefrontal and parietooccipital brain areas have been shown to reflect a functional mechanism to increase post-error cognitive control and sensory attention (Cavanagh et al., 2009; Cohen et al., 2009; Cohen and Cavanagh, 2011) respectively. Thus, PLVs were calculated for all channel combination and all frequencies in the time window from −200 and 1000 ms. Then, connectivity measure between mid-frontal (electrodes FC1, FCz, FC2, C1, Cz, C2), lateral prefrontal (electrodes F3, F5, F4, F6), and parietooccipital (electrodes PO7, PO3, POz, PO4, PO8, O1, Oz, and O_2_) scalp regions were extracted for each participant in three separate time windows, i.e., 200–400, 400–600, and 600–800 ms. Dependent-samples *t* tests were conducted to test any difference between conditions (C vs I). Differences between groups (LA+ vs LA– vs H) were estimated by means of a between-subject ANOVA, using groups (LA+ vs LA– vs H) as main factor and the differences between incorrect and correct condition (I minus C) as dependent measures.

Finally, the relation between signs and symptoms of LA and brain markers of error monitoring was investigated by means of a multiple linear regression model predicting error-related band power changes from LA phenotypes (LA+, LA–), TULIA scores (normalized in *z* scores), total brain lesioned volume (c^3^ normalized in *z* scores) and the Frontal Assessment Battery (FAB) scores (normalized in *z* scores); i.e., Y_i_ = β_0_ + β_i_X_i + interactions terms+_ ε_I_. Data of all the patients (LA+ and LA–) were included in the linear model, thus allowing to test which of the main predictors or their interaction terms, predicted error-related EEG dynamics. The brain lesioned volume and the FAB scores were chosen in the regression model to control for two clinically relevant indices that could account for by the variance between the three groups of patients, namely, any structural difference between patients’ brain and any difference in executive abilities. In keeping with the time-frequency analyses, power spectra in R and L condition were averaged, and the difference between incorrect minus correct condition was obtained. From these obtained values, β coefficients for the main effects and the interactions terms, and their *p*-values were calculated for each electrode and each time (from 500 to 1000 ms)-frequency (from 4 to 30 Hz) point across the whole patients’ sample.

## Results

### Time-domain analysis

**Figure 3.**
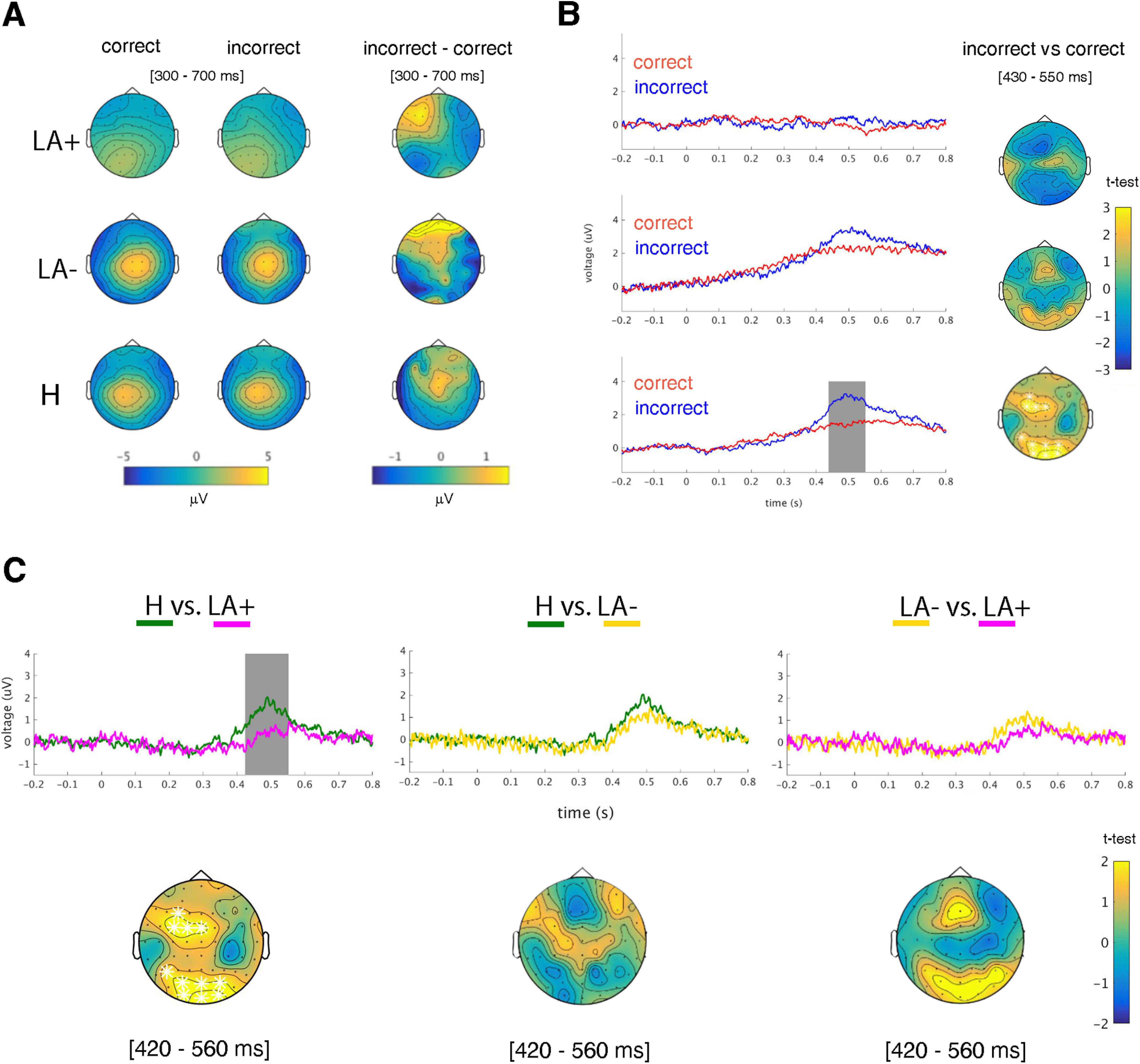
ERPs analysis. ***A***, Topographical maps of the early oPe in the time range 300–700 ms, for each group (LA+, LA–, and H) and each condition (correct and incorrect), and for the difference incorrect minus correct condition. ***B***, Time course of early oPe for each group (LA+, LA–, and H) in correct (red) and incorrect (blue) condition at the significant frontocentral cluster of electrodes (i.e., FC1, FCz, FC2, C1). The gray box highlights significant time points at which early oPe voltage differs between incorrect versus correct condition. Right-ward topographical maps show the significant frontocentral cluster (white markers) resulting from the contrast between incorrect minus correct condition, for each group (LA+, LA–, and H) in the time range 430–550 ms. ***C***, Time course of early oPe (upper-row) for each group (LA+, LA–, and H) at the mid-frontal cluster. The gray box highlights significant time points in which early oPe voltage differs between groups (H vs LA+, H vs LA–, and LA– vs LA+). Lower-row shows significant mid-frontal and parietooccipital clusters (white markers) resulting from the contrast between groups (H vs LA+, H vs LA–, and LA– vs LA+) in the time range 420–560 ms.

Permutation tests resulting from the contrast between incorrect versus correct conditions revealed significant positive clusters only for H (Fig. 3*A*). In particular, a significant voltage increase was found in incorrect trials in the 430- to 550-ms time window, at a mid-frontal (t-max: 2.74, *p* < 0.001, electrode FCz; Fig. 3*B*) and occipital (t-max: 3.27, *p* < 0.001, electrode Oz) cluster. No negative cluster was found from this analysis. The independent-samples *t* tests conducted between groups (LA+ vs LA–, LA+ vs H, LA– vs H; Fig. 3*C*) revealed positive clusters only for the contrast between H and LA+. In this, H exhibited increased voltage in the time window from 420 to 560 ms at mid-frontal (t-max: 2.36, *p* < 0.001; electrode FC3) and parietooccipital (t-max: 3.01, *p* < 0.001, electrode Oz) clusters.

### Time-frequency domain analysis

**Figure 4.**
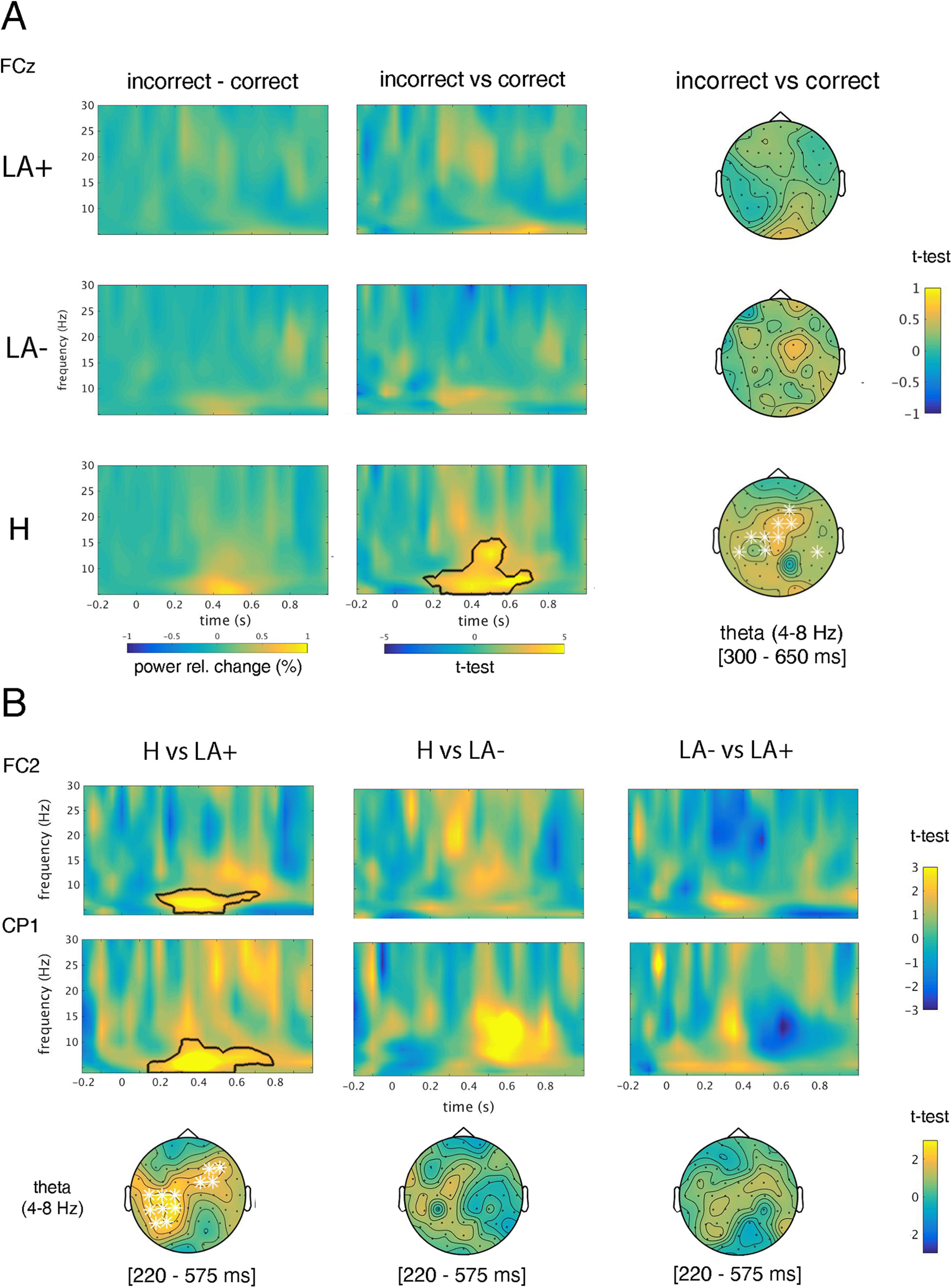
Time-frequency analysis. ***A***, Theta band-power differences (black contour line) resulting from the contrast between incorrect and correct condition for each group (LA+, LA–, and H) along the whole frequency spectrum (from 4 to 30 Hz). Right-ward topographical maps depict a cluster of electrodes (white asterisks) in which theta band-power activity differs between incorrect versus correct condition (time window from 300 to 650 ms). ***B***, Upper-row shows statistical differences of theta band-power activity resulting from the contrast between groups (H vs LA+, H vs LA–, and LA– vs LA+). The bottom row depicts significant clusters of electrodes in which theta band-power activity (4–8 Hz) differs between groups (white asterisks).

As for ERPs, the contrast between incorrect versus correct conditions revealed significant clusters only for the H group. More specifically, a significant increase of theta-band (4–8 Hz) was found in incorrect trials in the time range running from 300 to 650 ms at a mid-frontal cluster (t-max: 4.78, *p* < 0.001, electrode FCz; Fig. 4*A*). The independent-samples *t* tests between groups (LA+ vs LA– vs H; Fig. 4*B*) revealed positive clusters only for the contrast H versus LA+, accounted for by the fact that H exhibited increased theta power in the time range 420–575 ms at mid-frontal (t-max: 2.39, *p* < 0.001; electrode FC1) and parietooccipital (t-max: 2.74, *p* < 0.001, electrode CP1) clusters.

### Connectivity analysis

#### Mid-frontal to lateral-frontal connectivity

**Figure 5.**
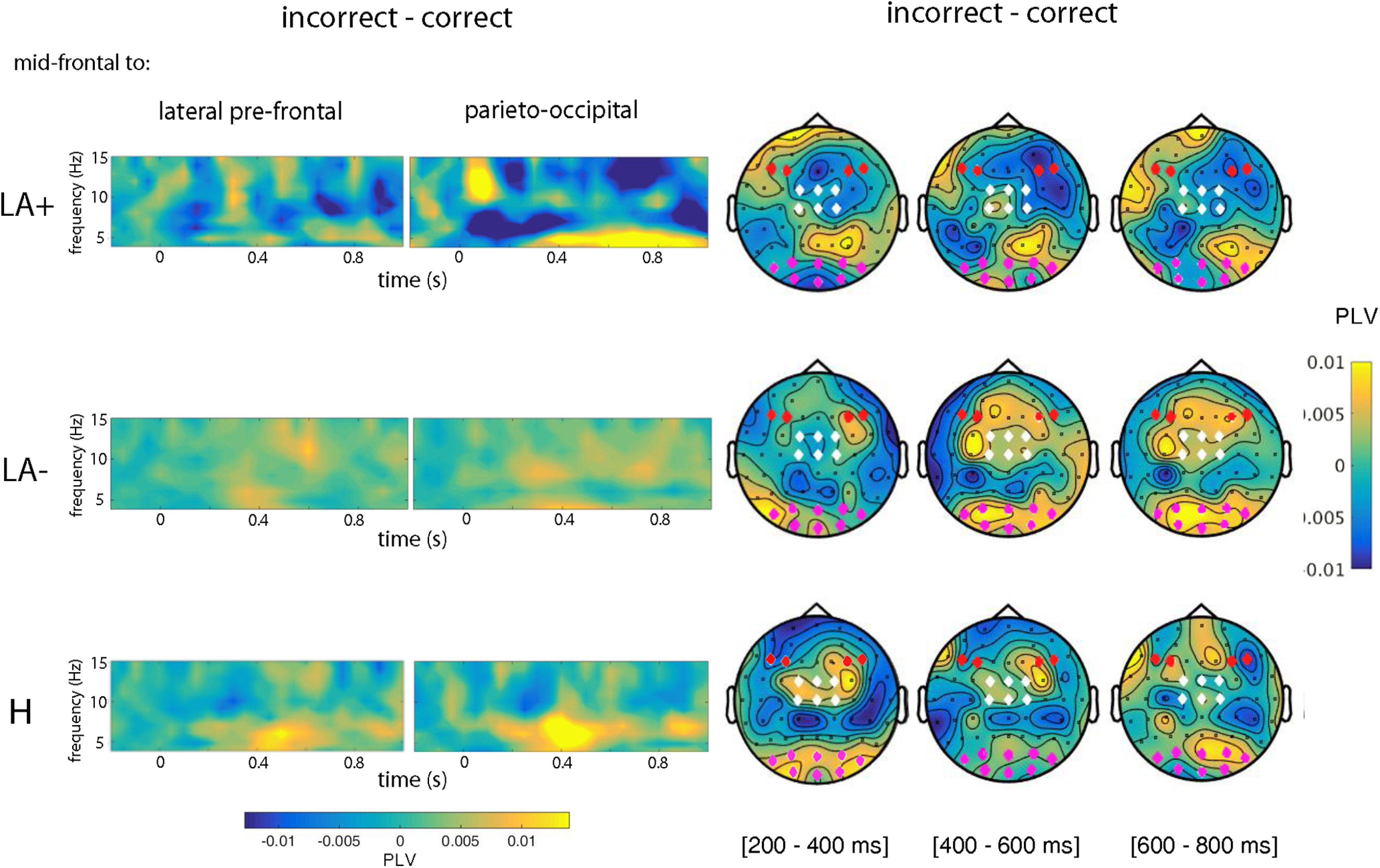
Phase connectivity analysis. Theta phase connectivity between mid-frontal (FC1, FCz, FC2, C1, Cz, C2), parietooccipital (PO7, PO3, POz, PO4, PO8, 01, 0z, O_2_), and lateral prefrontal electrodes (F6, F4, F3, F5), for each group (LA+, LA–, and H). Values refer to the difference between incorrect and correct condition and are plotted from 4 to 15 Hz for visualization purposes. Topographical maps depict theta connectivity between mid-frontal (white diamonds), lateral prefrontal (red diamonds), and parietooccipital electrodes (violet diamonds) in three time windows (200–400, 400–600, and 600–800 ms), for each group (LA+, LA–, and H).

The dependent-samples *t* tests conducted between incorrect versus correct condition revealed significant effects only for H (*t* = 2.18, *p* < 0.016) in the time window from 400 to 600 ms. The effect was explained by an increased theta phase connectivity for the observation of incorrect actions (Fig. 5, left panel). No further significant effect was found in any other time windows. The significant effect of the between-subjects ANOVA (*F*_(2,43)_ = 5.43, *p* < 0.01) was explained by a lower theta phase connectivity in LA+ (mean: –0.02, range: –0.01–0.05) with respect to both LA– (mean: 0.04, range: –0.1–0.16; *p* < 0.05) and H (mean: 0.05, range: –0.05–0.26; *p* < 0.001) in the same time range (i.e., 400–600 ms). No further effect was found.

#### Mid-frontal to parietooccipital connectivity

The dependent-samples *t* tests computed between incorrect versus correct condition revealed multiple significant effects. An increased error-related theta phase synchronization was found for both LA– (*t* = 2.53, *p* < 0.02) and H (*t* = 2.68, *p* < 0.01) in the time window from 200 to 400 ms. This effect remained significant also in the subsequent time window (i.e., 400–600) only for H (*t* = 2.64, *p* < 0.02). No significant effect was found in the time window from 600 to 800 ms. The significant effect of the between-subjects ANOVA (*F*_(2,43)_ = 3.91, *p* < 0.02) was explained by a decreased theta phase connectivity in LA+ (mean: –0.01, range: –0.01–0.03) with respect to both LA– (mean: 0.06, range: –0.12–0.15; *p* < 0.03) and H (mean: 0.04, range: –0.07–0.20; *p* < 0.05) from 200 to 400 ms. No further significant effect was found.

### Predictive estimates of TULIA scores on frontal theta power

**Figure 6.**
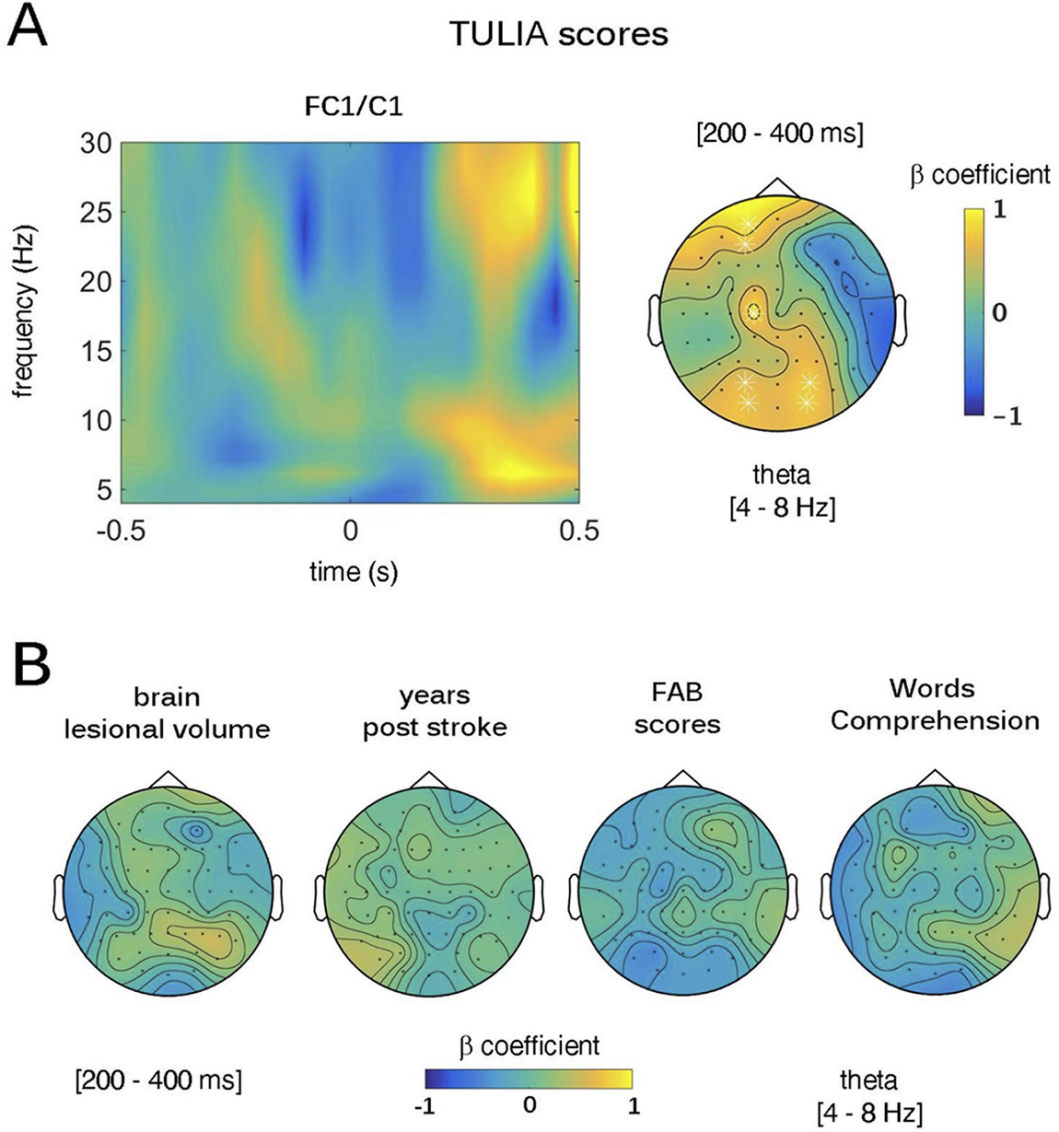
Link between apraxic phenotypes and mid-frontal theta oscillations. Multiple linear regression between Test of upper limb apraxia (TULIA) scores, brain lesioned volume, Frontal Assessment Battery (FAB) scores, days after stroke, word comprehension, and power spectra. ***A***, Main effect of TULIA scores; left column displays β coefficients in the time-frequency space over the significant cluster of electrodes (FC1/C1); right column depicts the relation between theta-band power and TULIA scores in the time window running from 200 to 400 ms. ***B***, Nonsignificant effect of brain lesion volume (left column), years after stroke (central-left column), FAB scores (central right column), and words comprehension (right column) on theta-band power in the time windows from 200 to 400 ms.

The linear regression model revealed a significant main effect of the TULIA test (*F*_(12,5)_ = 3.2, *p* < 0.05, *r*^2^ = 0.72, *r*^2^ adjusted = 0.67) over a frontocentral cluster of electrodes (FC1, C1). More specifically, we found a significant direct relation (β = 0.85, *p* < 0.01) between theta power and TULIA scores in the time range 200–400 ms (Fig. 6*A*). No other main effect nor interaction were found for the other predictors (i.e., brain lesion volume, days after stroke, FAB scores, and words comprehension; Fig. 6*B*) within the same time window at that electrode site.

Descriptive statistics shows that S2 were the most prevalent errors (mean = 12; SD = 4.69), followed by S0 (mean = 4.83; SD = 3.25), S1 (mean = 4.33; SD = 1.03) and S3 (mean = 1; SD = 9.89). S2 errors refer to a difficulty of apraxic patients to correct the trajectory of a gesture, and committing errors without correction. S0 errors refer to severe problems in executing the movement, and S1 index problems in both trajectory and semantic content of the movement. S3 errors (the least frequent) include the correction of ongoing movements.

### Tract disconnection probability

Tract disconnection probability (mean, standard deviation, and number of patients for each group that showed >0.5 probability of disconnection) for both LA+ and LA– are shown in Table 6; *t* test comparison with false discovery rate correction for multiple comparisons did not show significant differences between groups.

### Subjective reports of virtual embodiment

Table 3 reports average ownership and vicarious agency ratings in LA+, LA–, and H. Individual ratings were entered in a mixed-design ANOVA with GROUP (LA+, LA–, H) as between-subjects factor, and EMBODIMENT (two levels: Ow vs Ag), ACCURACY (two levels: C vs I) and LIMB (two levels: R vs L) as within-subjects factors. Newman–Keuls *post hoc* test was adopted for multiple comparisons. The ANOVA resulted in a significant main effect of the ACCURACY (*F*_(1,19)_ = 7.6, *p* < 0.02, η^2^ = 0.28), explained by overall higher values of embodiment for C (mean ± SD = 0.61 ± 0.25) with respect to I (mean ± SD = 0.56 ± 0.25) actions. No further significant main effect nor interaction were found (all *p*s > 0.15). Moreover, subjective scores of embodiment did not correlate with any of the error-related EEG signals, namely, oPe amplitude and theta-band activity (for Ow: LA+ = all *p*s > 0.2, LA– = all *p*s > 0.05, H = all *p*s > 0.07; for Ag: LA+ = all *p*s > 0.5, LA– = all *p*s > 0.1, H = all *p*s > 0.07).

**Table 3:** Subjective ratings of ownership and agency

	Ownership				Vicarious agency			
	Right		Left		Right		Left	
	Correct	Incorrect	Correct	Incorrect	Correct	Incorrect	Correct	Incorrect
LA+	0.57 ± 0.40	0.58 ± 0.38	0.60 ± 0.40	0.61 ± 0.39	0.58 ± 0.38	0.58 ± 0.36	0.59 ± 0.41	0.58 ± 0.41
LA–	0.44 ± 0.28	0.41 ± 0.28	0.48 ± 0.29	0.38 ± 0.31	0.45 ± 0.28	0.43 ± 0.28	0.48 ± 0.30	0.38 ± 0.32
H	0.37 ± 0.23	0.29 ± 0.20	0.35 ± 0.21	0.27 ± 0.20	0.36 ± 0.24	0.25 ± 0.15	0.33 ± 0.22	0.25 ± 0.18

Each cell contains the mean ± SD of the mean for each condition and each group.

## Discussion

We explored in left brain-damaged people with or without apraxia, and in a control group of healthy individuals (H) the electrocortical dynamics of error observation by combining immersive virtual reality and EEG recording. Results in the time and time-frequency domain showed that observation of erroneous actions brought a suppression of early oPe and theta activity in LA+ and LA–. In addition, LA+ showed a significant difference when compared with H, that was not shown when H were compared with LA–, suggesting an impairment in error processing for LA+. In addition, LA+ highlighted aberrant theta phase synchronicity between frontofrontal and frontoparietal networks, with respect to both LA– and H. To the best of our knowledge, this study reports the first evidence of altered performance monitoring in patients with LA. Based on the theoretical framework of the conflict monitoring theories (Botvinick et al., 2001; Yeung et al., 2004) and of the affordance competition hypothesis (Cisek, 2007; Pezzulo and Cisek, 2016), we submit that this impairment could be driven by the LA patients’ original difficulty in selecting the appropriate action schema to implement goal-directed behaviors, and in suppressing inappropriate conflicting affordances arising from the observation of an object. Consequently, the excessive burden of unresolved conflict prevents patients from fluid action understanding and impairs the EEG dynamics that underpin appropriate performance monitoring.

The absence of the early Pe in the group of LA+ when compared with H provides novel evidence in support of our hypothesis. Early Pe is a P300-like positive-going component that differentiates from late Pe (Falkenstein et al., 2000) for maximally peaking over mid-frontal electrodes in error trials (Ullsperger et al., 2014), and for originating from mid-frontal cortical sources (Van Boxtel et al., 2005). Also, early Pe dissociates from the late Pe in terms of functional significance. In keeping with P300 event-related brain potential theories (Polich, 2007), early Pe seems to resamble the activity of a task-related, frontal cognitive control mechanism associated to automatic error processing (prediction errors or mismatch), whereas late Pe may be linked to higher-order processes, like memory processing or affective reactions to maladaptive/infrequent stimuli or internal model updating and potential adjustments (Falkenstein et al., 2000; Di Gregorio et al., 2018). In the present study, LA+ did not show the classical early Pe following incorrect trials; LA– did not show a difference between incorrect or correct actions. However, one can qualitatively appreciate how LA– showed a modulation in the time series of the ERP, that is not visible in the LA+; also, when contrasts between groups are performed, H showed a significant difference as compared with LA+, but not when compared with LA–. This suggests a reduced responsivity of LA+ performance monitoring system that interferes with the resolution of the conflict generated from the competition between incorrect action outcomes and correct action schema (Botvinick et al., 2001; Yeung et al., 2004). Interestingly, studies demonstrate that P300-like waveforms originate from phasic activity of the norepinephrine system and may underlie the learning processes responsible for subsequent motor improvement (Nieuwenhuis et al., 2005; Dayan and Yu, 2006). Therefore, the absence of early Pe in LA+, may not only index a defective conflict processing, but also an impaired ability to implement flexible behavioral adaptation in a cascade-like sequence of neurocognitive events. Another relevant result of our study is the absence of the oERN across all the subjects and experimental groups. Previous studies using virtual-reality (Pavone et al., 2016; Pezzetta et al., 2018; Spinelli et al., 2018) or other methods (van Schie et al., 2004; Bates et al., 2005; Koban et al., 2010; de Bruijn and von Rhein, 2012), reported that observation of others’ action errors evoked an oERN in the onlookers’ brain. Here, oERN suppression can be explained in terms of an age-dependent effect (Gehring and Knight, 2000; Nieuwenhuis et al., 2001; Mathewson et al., 2005), or in view of the novel evidence that errors can elicit error-positivity in the absence of an ERN (Di Gregorio et al., 2018; Pezzetta et al., 2021). While our results fit adequately with the above options, drawing firm conclusions is likely complicated by the original aim of this study and the characteristics of the sample. Absence of oERN was admittedly unexpected; therefore, future works should tackle this important issue using *ad hoc* developed experimental designs.

Analyses of brain oscillatory activity provide another important support for altered performance monitoring in apraxia. Indeed, our results indicate a significant error-related suppression of mid-frontal theta power in the group of LA+. Cognitive control over goal-directed behavior is a highly flexible process that integrates information coming from the actual context and specific task-related demands (Helfrich and Knight, 2016). A large-scale network governed by the prefrontal cortex and composed by distant and yet functionally related cortical and subcortical areas (Miller and Cohen, 2001), rhythmically orchestrates such integration. Electrophysiology evidence demonstrates that activity in the prefrontal cortex becomes significantly higher when deviant outcomes (Dürschmid et al., 2016) or errors (Fonken et al., 2016) are detected. EEG studies in nonhuman primates also demonstrate that this multiplexed computational activity is conducted in distinct frequency bands, time and brain (scalp) locations (Akam and Kullmann, 2014). Notably, in humans, an increase of mid-frontal theta power underlies error execution (Trujillo and Allen, 2007; Hanslmayr et al., 2008; Cavanagh et al., 2009, 2012; Munneke et al., 2015) and error observation (Pavone et al., 2016; Pezzetta et al., 2018; Spinelli et al., 2018). This effect has been convincingly associated to conflict processing and resolution (Cohen, 2014). Together with time-domain results, the suppression of mid-frontal theta power in LA+ patients suggests that conflict arising from the competition between correct and incorrect action schema is not adequately resolved in the patients’ performance monitoring system. Moreover, connectivity analyses show a decreased theta synchronicity between frontofrontal and frontoparietooccipital areas in LA+ with respect to both LA– and H. Phase synchronicity reflects a coherent burst of activity of neuronal populations in distant cortical regions. Such an alignment of brain oscillatory dynamics in time facilitates the communication between networks and ultimately enables efficient cognitive processing (Voloh et al., 2015; Daitch et al., 2013). Tellingly, frontofrontal and frontoparietal network dynamics has been suggested to play a crucial role in making fluid cognitive control (Gregoriou et al., 2009; Nácher et al., 2013; Phillips et al., 2014). EEG studies show that posterior theta phase enhancement in these networks underlies perceptually integration of maladaptive information, and represents a call to increase cognitive control for subsequent behavioral adjustment (Cavanagh et al., 2009; Cohen et al., 2009; Cohen and Cavanagh, 2011). That LA+ patients exhibit aberrant oscillatory patterns during action monitoring, suggests not only a reduced capacity of their performance monitoring system to resolve the conflict, but also a difficulty to capitalize on perceptual and sensorimotor information flow from action observation. This latter claim fits with previous reports showing that motor skills of apraxic patients may influence their visual action understanding, and vice versa (Pazzaglia et al., 2008a).

It should be noticed that we found no difference between correct and incorrect actions in LA+ and LA– in terms of theta and Pe signals absolute values. However, further contrasts between groups, obtained from incorrect minus correct actions, showed a significant difference between LA+ and H, but not between LA– and H, thus highlighting how H showed increased theta activity in response to errors, that was instead not found in LA+. The lack of a direct difference when comparing LA+ and LA– might be because of lack of sensitivity to pick up differences between patients’ groups because of the reduced sample. Tellingly, however, connectivity analyses in the theta range show that LA+ had lower theta as compared with both LA– and H both in the frontal and parietal regions, suggesting an impaired error-monitoring process in LA+. Another result that deserves discussion concerns the extent to which altered performance monitoring parallels the apraxic phenotypes. This was tested by means of a multiple linear regression model, predicting theta power activity from an index of the apraxic impairment (TULIA scores) and two other main factors that significantly differed between LA+ and LA–, i.e., lesioned brain volume and an index of general functionality of frontal lobes (FAB scores). Results show evidence for a direct relation between the severity of apraxia and error-related mid-frontal theta power, so that reduced error-related mid-frontal theta power was predicted by the severity of the disease (indexed by lower TULIA scores). This effect hints at the close link between the apraxic phenotype and the integrity of the performance monitoring system and confirms our hypothesis that symptoms of apraxia prevent patients’ ability to resolve the conflict generated by the observation of incorrect actions, regardless of the amount of lesioned cortical volume and of the patients’ impairment in frontal executive functions, as indexed by FAB scores. Table 4 and Table 5 report lesion data of LA+, LA− and the results of lesions subtraction (LA+ minus LA−). The lesion mapping data suggest that lesions to inferior frontal gyrus, rolandic operculum, insula, and putamen, as well as to superior frontooccipital and superior longitudinal fasciculi seem to differentiate the two groups. These patterns of results are in line with previous findings showing how LA+ exhibit behavioral deficits during prediction, gesture comprehension and error detection tasks (Kilner, 2011; Avenanti et al., 2013; Keysers and Gazzola, 2014; Urgesi et al., 2014). Moreover, the most significant difference between the two groups is represented by the involvement of the basal ganglia (i.e., putamen) and the insula in LA+ versus LA–. Crucially, these regions have been found to play a role in error detection and performance monitoring (Falkenstein et al., 2001; Klein et al., 2007; Yang et al., 2015). Importantly, the superior frontooccipital fasciculus and superior longitudinal fasciculus were also lesioned in the LA+ group, thus supporting the hypothesis that deficits in our apraxic patients might have been because of the association between frontotemporal, frontoparietal, and basal ganglia lesions.

**Table 4:** Lesion overlap in LA+ and LA–

Area	Number of lesioned voxels	% of lesioned voxels	MaxX	MaxY	MaxZ
LA+					
Frontal_Inf_Oper_L	1169	14	−36	5	23
Frontal_Inf_Tri_L	2048	10	−40	21	−1
Rolandic_Oper_L	3453	43	−45	−10	22
Insula_L	8349	55	−39	−9	24
Putamen_L	1348	17	−31	10	−1
Heschl_L	103	6	−47	−11	3
Anterior_limb_of_int	541	17	−26	7	17
Anterior_corona_rad	3228	47	−28	11	20
Posterior_corona_rad	750	20	−30	−31	26
Superior_corona_rad	4647	62	−29	−2	19
External_capsule_R	2146	38	−32	9	−1
Superior_longitudina	2936	44	−33	−3	21
Superior_fronto-occi	356	70	−24	4	19
LA–
Rolandic_Oper_L	452	6	−46	−1	6
Postcentral_L	2108	7	−66	−14	14
SupraMarginal_L	1710	17	−67	−26	26

**Table 5:** LA+ and LA– subtraction lesion map

Subtraction 6 LA+ minus 6 LA– (lesioned voxels in at least 3 patients)					
Area	Number of lesioned voxels	% of lesioned voxels	MaxX	MaxY	MaxZ
Frontal_Inf_Oper_L	786	9	−36	5	23
Frontal_Inf_Tri_L	2025	10	−40	21	−1
Rolandic_Oper_L	1636	21	−42	−2	12
Insula_L	7392	49	−37	−9	24
Putamen_L	1348	17	−31	10	−1
Anterior_limb_of_int	541	17	−26	7	17
Anterior_corona_radi	2976	43	−28	11	20
Superior_corona_radi	4267	57	−29	−2	19
Posterior_corona_rad	750	20	−30	−31	26
External_capsule_R	2146	38	−32	9	−1
Superior_longitudina	2784	42	−33	−3	21
Superior_fronto-occi	351	69	−24	4	19

**Table 6:** Probability of tract disconnection for LA+ and LA– patients

	LA+		LA–	
	Mean	SD	Mean	SD
Anterior_Commissure	0.44	0.42 (2)	0.13	0.2 (0)
Anterior_Thalamic_Projections_Left	**1**	0 (6)	**0.74**	0.32 (2)
Arcuate_Anterior_Segment_Left	**0.99**	0.02 (6)	0.43	0.46 (2)
Arcuate_Long_Segment_Left	**1**	0 (6)	**0.56**	0.39 (3)
Arcuate_Posterior_Segment_Left	**0.7**	0.37 (4)	0.35	0.39 (3)
Cingulum_Left	**0.84**	0.29 (5)	0.45	0.42 (3)
Cingulum_Left_anterior	**0.66**	0.43 (4)	0.36	0.44 (2)
Corpus_callosum	**1**	0 (6)	**0.9**	0.15 (6)
Cortico_Spinal_Left	**1**	0 (6)	**0.72**	0.38 (5)
Face_U_tract_Left	**0.6**	0.22 (4)	0.26	0.4 (2)
Fornix	0.45	0.4 (3)	0.17	0.32 (1)
Frontal_Aslant_Tract_Left	**1**	0 (6)	**0.88**	0.26 (5)
Frontal_Commissural	**1**	0.01 (6)	**0.6**	0.49 (4)
Frontal_Inferior_longitudinal_Left	**0.89**	0.1 (6)	0.34	0.38 (3)
Frontal_Orbito_Polar_Left	**0.79**	0.39 (5)	0.2	0.38 (1)
Frontal_Superior_Longitudinal_Left	**0.65**	0.5 (4)	0.41	0.4 (3)
Fronto_Insular_tract1_Left	0.13	0.07 (0)	0.02	0.06 (0)
Fronto_Insular_tract2_Left	0.31	0.04 (0)	0.08	0.12 (0)
Fronto_Insular_tract3_Left	**0.68**	0.06 (6)	0.35	0.38 (3)
Fronto_Insular_tract4_Left	**0.98**	0 (6)	0.41	0.48 (3)
Fronto_Insular_tract5_Left	**0.94**	0.08 (2)	0.38	0.47 (2)
Fronto_Marginal_tract_left	0.36	0.42 (2)	0.05	0.13 (0)
Fronto_Striatal_Left	**1**	0 (6)	**0.85**	0.2 (2)
Handinf_U_tract_Left	**0.87**	0.13 (6)	0.3	0.47 (2)
Handmid_U_tract_Left	0.17	0.19 (0)	0.17	0.19 (0)
Handsup_U_tract_Left	0.49	0.53 (2)	0.48	0.53 (3)
Inferior_Fronto_Occipital_fasciculus_Left	**0.99**	0.02 (2)	0.45	0.51 (3)
Inferior_Longitudinal_Left	0.45	0.44 (2)	0.16	0.40 (1)
Optic_Radiations_Left	0.35	0.43 (2)	0.05	0.12 (0)
Paracentral_U_tract_Left	0.03	0.08 (0)	0	0 (0)
Pons_Left	**1**	0 (6)	**0.76**	0.3 (5)
Superior_Londgitudinal_Fasciculus_III_Left	**1**	0 (6)	**0.77**	0.26 (5)
Superior_Londgitudinal_Fasciculus_II_Left	**0.99**	0.03 (6)	**0.65**	0.5 (4)
Superior_Londgitudinal_Fasciculus_I_Left	**0.83**	0.31 (5)	**0.54**	0.45 (4)
Uncinate_Left	**0.86**	0.29 (5)	0.37	0.43 (3)

For a given lesion, Tractotron provides a probability of disconnection for almost all known tracts (Foulon et al., 2018). The probability corresponds to the lesioned voxel with the highest % value; therefore, patients with a probability of disconnection >50% (=0.5) are usually considered as disconnected. Values of 1 indicate maximal probability of tract disconnection. Tracts that exceed the 50% of probability of disconnection are shown in bold. The table shows for each tract the mean value, the standard deviation and the number of patients that exceed the 0.5 probability of disconnection, for each group.

A final point of discussion concerns the analysis of subjective reports. In keeping with previous studies (Padrao et al., 2016; Pavone et al., 2016) embodiment scores were lower during observation of erroneous with respect to correct actions. However, here we did not find any relation between error-related EEG signatures and subjective reports of embodiment, neither in healthy (H) nor in brain-damaged individuals (LA+ and LA–). One possible explanation may be due to collecting embodiment ratings (ownership and vicarious agency) only in the 25% of trials which, combined with the small sample size may have determined this lack of sensitivity. Alternatively, and in keeping with previous report (Spinelli et al., 2018), one may note that the relation between virtual embodiment and error-related brain signatures is merely correlative and not causative. Future work is needed to understand whether inducing embodiment of artificial (virtual) upper limbs might play any specific role in improving the action monitoring capacity in people suffering from higher-order motor disorders. The issue of patients’ sample size deserves discussion. Indeed, LA+ group and LA– count a relatively small number of individuals. This is mainly because of the adoption of very restrictive inclusion criteria based on socio-demographic data, brain-injury site, and individuals’ compliance to our EEG protocol in virtual reality. Therefore, while on the one hand the selection criteria reduced the sample size, on the other it prevented us from recruiting a nonhomogeneous patients’ sample and jumping to misleading conclusions. However, future studies with larger cohorts of patients are recommended to replicate these results. Furthermore, we maintained the unbalance of frequency of occurrence typical of error studies by including 48 incorrect trials and 72 correct ones. Previous methodological studies have shown that increasing the number of trials does not affect the reliability of error signatures and a minimum of 8 trials may be sufficient to reliably elicit ERN and Pe (Olvet and Hajcak, 2009; Pontifex et al., 2010). In conclusion, our results indicate reduced electrocortical activity of the performance monitoring systems in brain damaged patients with LA+ suggesting that ideomotor LA brings about difficulties in error processing when observing the actions of others. Our paradigm paves the way to potentially interesting new studies on the role that theta-band oscillatory entrainment over prefrontal cortices may play in facilitating patients’ performance monitoring. Moreover, our study casts fresh light on the neuro-cognitive architecture characterizing apraxia and thus has the potential to inspire novel rehabilitation protocols.

## Synthesis

Reviewing Editor: Christopher Colwell, UCLA School of Medicine Decisions are customarily a result of the Reviewing Editor and the peer reviewers coming together and discussing their recommendations until a consensus is reached. When revisions are invited, a fact-based synthesis statement explaining their decision and outlining what is needed to prepare a revision will be listed below. The following reviewer(s) agreed to reveal their identity: Quentin Moreau.

The authors did a good job revising the ms. The reviewers are satisfied with the revised ms.
